# Association between perceived discrimination and depressive symptoms among male and female mining workers in Chile: a sex-stratified analysis and the mediating role of psychological distress

**DOI:** 10.1186/s12889-025-25787-2

**Published:** 2026-01-21

**Authors:** Gonzalo Bravo-Rojas, Ignacio Castelluci, Alejandra Fuentes-García, José Ignacio Méndez

**Affiliations:** 1 Programa de Doctorado en Salud Pública, Facultad de Medicina, Universidad de Chile, Santiago, Chile; 2 Centro de Estudio del Trabajo y Factores Humanos, Facultad de Medicina, Universidad de Valparaíso, Valparaíso, Chile; 3 Escuela de Salud Pública, Facultad de Medicina, Universidad de Chile, Santiago, Chile; 4 Departamento de Medicina Familiar, Facultad de Medicina, U. Católica de Chile, Santiago, Chile; 5 Programa Medicina del Trabajo y del Ambiente, U. San Sebastián, Valdivia, Chile

**Keywords:** Workplace discrimination, Depressive symptoms, Psychological distress, Mining workers, Occupational health.

## Abstract

**Background:**

Workplace discrimination is a recognised social determinant of mental health. However, evidence regarding its impact in highly masculinised and demanding industries, such as mining, remains limited, particularly in Latin America. This study aimed to estimate the association between perceived and observed workplace discrimination and depressive symptoms among mining workers in Chile, and to evaluate the mediating role of psychological distress.

**Methods:**

A cross-sectional study was conducted among 712 employees from a large-scale mining company, including both principal and subcontracted workers. Data were collected via an online survey between September and December 2024. Depressive symptoms were assessed using the Patient Health Questionnaire-9 (PHQ-9), and psychological distress was measured with the Kessler Psychological Distress Scale (K6). Logistic regression models, stratified by sex, were used to assess associations. Causal mediation analysis was conducted within a counterfactual framework to decompose the total effect of workplace discrimination into direct and indirect effects through psychological distress.

**Results:**

The prevalence of perceived workplace discrimination was 13.56%, observed discrimination was 19.29%, and moderate/severe depressive symptoms were present in 8.31% of participants. Perceived workplace discrimination was associated with significantly higher odds of depressive symptoms (adjusted OR = 5.17; 95% CI: 2.70–9.91). Similar associations were found for observed discrimination (adjusted OR = 4.01; 95% CI: 2.20–7.31). Sex-stratified logistic regression analyses indicated that these associations were stronger among men than among women. Causal mediation analysis demonstrated that psychological distress mediated a substantial proportion of the association, accounting for 81.4% of the total effect for perceived discrimination and 65.9% for observed discrimination.

**Conclusions:**

Workplace discrimination is a significant risk factor for depressive symptoms among mining workers, operating largely through increased psychological distress. These findings highlight the need for organisational interventions that address both discriminatory practices and their psychological consequences, particularly in male-dominated industries such as mining.

**Supplementary Information:**

The online version contains supplementary material available at 10.1186/s12889-025-25787-2.

## Highlights

Workplace discrimination is strongly associated with depressive symptoms in mining workers.

Psychological distress mediates most of the discrimination-depression relationship.

Men show stronger associations between discrimination and depressive symptoms than women.

Observing discrimination also increases workers’ risk of depression.

Organisational policies must address both discriminatory practices and emotional consequences.

**Supplementary Information:**

The online version contains supplementary material available at 10.1186/s12889-025-25787-2.

## Introduction

Extensive evidence indicates that perceived discrimination is a significant predictor of mental health deterioration. One study reported that 38% of 18,000 participants from nine countries of the former Soviet Union reported experiencing moderate or severe discrimination, significantly increasing the likelihood of psychological distress [1]. Similarly, a prospective cohort study demonstrated that perceived discrimination is a significant predictor of depression among older adults in the United States [2]. Research conducted in Europe revealed that when discrimination occurs for multiple reasons, depressive symptoms increase proportionally, particularly in countries with lower economic development [3]. Furthermore, the accumulation of discriminatory experiences over time has been shown to increase the risk of mental health problems by 46%, regardless of socioeconomic status [4]. Additionally, the specific vulnerability of traditionally marginalised groups, such as African American men, confirms that daily perceived discrimination exacerbates depressive symptoms [5].

Although the detrimental effects of discrimination on mental health have been well established, its influence also extends to physical health domains. A meta-analysis revealed negative associations between perceived discrimination and physical health, primarily attributed to stress responses and unhealthy behaviours induced by discriminatory experiences [6]. Moreover, repeated discrimination has been shown to significantly increase the risk of all-cause mortality, especially among socially vulnerable groups such as immigrants and individuals under financial stress [7].

In this context, the workplace represents a critical environment where perceived discrimination can further exacerbate its harmful effects. A meta-analysis demonstrated that perceived workplace discrimination adversely affects both the mental and physical health of employees, with occupational stress acting as a key mediator [8]. Additionally, observed workplace discrimination appears to have an even stronger effect on workers’ mental health than does personally experienced discrimination [8]. A recent review reinforced the strength of this evidence, indicating that most studies investigating the association between perceived workplace discrimination and mental health problems, such as depression and stress, report negative outcomes [9]. This form of discrimination not only contributes to mental health problems but also negatively impacts occupational outcomes such as job satisfaction and work engagement [9]. Furthermore, factors such as sex, ethnicity, and age significantly influence perceptions of discrimination, with women and ethnic minorities being the most affected groups [9]. Although occupational stress has been identified as a mediator in the relationship between perceived workplace discrimination and mental health [8] and the mediating role of mental health has been highlighted in the association between repeated discrimination and mortality [7], few studies have employed causal mediation frameworks to disentangle how psychological mechanisms, particularly psychological distress, mediate the relationship between workplace discrimination and mental health outcomes.

Scientific evidence consistently shows a significant association between perceived workplace discrimination and depression, which affects various occupational groups and work settings, such as the healthcare sector [10, 11], firefighters [12, 13], armed forces [14], and other occupational sectors [15–18]. Several of these studies have also demonstrated a pronounced effect of workplace discrimination on women’s depression [11–13, 15, 16]. In addition to direct experiences, witnessing workplace discrimination has also been associated with adverse mental health outcomes, as it can contribute to a hostile work climate and collective stress responses among employees [8].

Despite the growing evidence linking perceived workplace discrimination and depression, to date, only one study in the mining sector has examined this relationship [19]. That study investigated the prevalence of workplace discrimination and harassment in an Australian mining company and reported that 28% of employees reported experiencing discrimination, which was significantly associated with a higher prevalence of depression, anxiety, and suicidal ideation [19]. Furthermore, the effects of discrimination on mental health persisted even after adjustments were made for demographic, occupational, and well-being variables [19]. To our knowledge, no prior studies have explored this association within the Latin American mining sector, making the present study one of the first to examine these dynamics in this critical economic context.

Thus, the mining sector represents a critical setting for addressing this gap. The copper mining industry in Chile is characterised by extreme working conditions that profoundly impact workers’ physical and mental health. This activity has been associated with diseases such as silicosis and musculoskeletal and cardiovascular disorders, as well as psychological issues such as chronic fatigue, stress, sleep disorders, and workplace violence, conditions that disproportionately affect women and subcontracted workers [20]. In this context, workplace harassment becomes a deeply rooted form of control within mining culture, where toughness is demanded, fear of exclusion is instilled, physical risk-taking is incentivised to meet targets, and women are marginalised. This creates a work environment where suffering is perceived as an unavoidable part of the job [21]. This mining labour culture, which is grounded in a form of defensive masculinity, severely affects workers’ mental well-being by fostering addictions, emotional disconnection, and the deterioration of family and social relationships [22].

Given this background, the present study has two objectives. First, we aimed to estimate the association between perceived workplace discrimination and depressive symptoms among employees of a large-scale mining company in Chile stratified by sex. Second, we aimed to evaluate the mediating role of psychological distress in the association between perceived workplace discrimination and depressive symptoms. In doing so, this research aims not only to address a gap in the literature but also to provide empirical evidence for the development of targeted interventions in a sector that is critical to the national economy.

## Methods

This study was conducted following the guidelines of the Strengthening the Reporting of Observational Studies in Epidemiology (STROBE) Statement [23] and the A Guideline for Reporting Mediation Analyses of Randomised Trials and Observational Studies (AGReMA Statement) [24]. and the Sex and Gender Equity in Research (SAGER) guidelines [25].

### Study design and sample

A cross-sectional study was conducted with a sample of workers from a large-scale mining company in Chile, including both direct employees of the principal company and subcontracted workers from external service providers. The sample size was estimated on the basis of a finite population of approximately 10,000 workers, assuming a 95% confidence level, a 5% margin of error, and an expected prevalence of 50%, achieving the minimum required sample size of 370 participants. Owing to the fluctuating and unregistered number of subcontractor employees, probabilistic sampling was not feasible, and a nonprobabilistic, convenience sampling approach was adopted instead. The final analytical sample included 712 participants, which exceeded the minimum required sample size and increased the study’s statistical power.

### Setting

The study was carried out between September and December 2024. Workers were invited to participate through two recruitment strategies. For employees of the principal company, invitations were sent via their institutional email, which included general information about the study and a link to the online questionnaire hosted on the SurveyMonkey platform. Access to institutional email addresses was authorised by the Human Resources Department of the principal company, following approval of the study protocol by the University Ethics Committee. In parallel, subcontracted workers received the same invitation via WhatsApp or email, which was delivered through their direct supervisors via contact details registered in the company system.

The survey comprises four sections: sociodemographic and occupational characteristics, depressive symptoms, psychological distress, and perceived workplace discrimination. The study protocol was approved by the Human Research Ethics Committee of the Faculty of Medicine, University of Chile (Project No. 128–2023, Record No. 118).

### Participants

Eligibility criteria included being an active employee of the large-scale mining company or an active subcontracted worker providing services to the principal company. All participants provided digital informed consent prior to beginning the survey, ensuring that ethical standards and participants’ understanding of the study were upheld.

### Variables

The primary variables of interest were depressive symptoms, psychological distress, and perceived workplace discrimination, measured as follows:

#### Depressive symptoms

Assessed via the Patient Health Questionnaire-9 (PHQ-9), a nine-item instrument previously validated [26]. Total scores range from 0 to 27, with scores of 5–9 indicating mild depression, scores of 10–14 indicating moderate depression, scores of 15–19 indicating moderately severe depression, and scores of 20 or more indicating severe symptoms. A cut-off score of ≥ 10 was used to dichotomise responses into moderate/severe (≥ 10) versus none/mild (< 10) depressive symptoms, which is consistent with prior literature [27].

#### Psychological distress

It was measured via the Kessler Psychological Distress Scale (K6), a six-item instrument previously validated, assessing the frequency of anxiety and depressive symptoms in the past month [28]. For the mediation analysis, psychological distress was treated as a continuous variable ranging from 0 to 24.

#### Perceived workplace discrimination

It was assessed via a self-administered questionnaire. It had been previously developed by the authors on the basis of a scoping review of the literature. The instrument evaluated both direct and observed experiences of discrimination in the workplace, as well as the perceived reasons for such incidents. Content validity was assessed by a panel of five experts with extensive experience in organisational psychology, social sciences, statistics, and mining. The panel included two PhDs in organisational behaviour and psychology with over 15 years of academic experience, one PhD in statistics with expertise in psychometric validation, and two senior professionals with over 20 years of experience in social sciences and human resource management within the mining sector. The questionnaire demonstrated adequate content validity through expert review, and a separate validation study has been accepted for publication [29], providing evidence of its reliability and construct validity. In the present study, perceived and observed workplace discrimination were measured as single-item, dichotomous indicators (yes/no). The full instrument and item coding are provided in Supplementary Material 1.

#### Covariates

A wide range of sociodemographic and occupational variables were collected to describe the sample, including age, sex (male/female), company type (principal or contractor, referring to whether the participant was directly employed by the main company or worked for an external service provider), job role (administrative, field, or both, referring to whether the participant primarily performed office-based tasks, operational fieldwork, or a combination of both), shift system (with or without any shifts), company tenure (< 5, 5–10, 11–20, > 20 years), mining industry tenure (< 5, 5–10, 11–20, > 20 years), parental status (yes/no), marital status (single, married, civil union, separated, divorced, widowed), educational level (primary/secondary, technical, university/postgraduate), and awareness of antidiscrimination policies (yes/no).

A subset of these variables was used in both the logistic regression and causal mediation models: age, number of children, educational level, shift work, job role, company type, and company tenure. This selection was guided by the analytical objective of estimating the association between perceived discrimination and depressive symptoms while controlling for factors theoretically and empirically associated with both the exposure and the outcome. Several of these variables, such as age, educational level and job role, have been commonly included in previous research examining workplace discrimination and mental health [10]. Other covariates, including number of children, shift work, company type and company tenure, were selected based on their relevance in the mining industry context and their potential influence on the experience of workplace discrimination and depressive symptoms. With respect to sex and gender identity, information was collected separately for sex (male/female) and gender identity (masculine, feminine, other). As only one participant identified as “other”, statistical analyses were conducted based on sex, using the categories male and female. Logistic regression models were stratified by sex to explore potential effect modification. In addition, sex was included as a covariate in the causal mediation models to adjust for confounding between the exposure (workplace discrimination), the mediator (psychological distress), and the outcome (depressive symptoms), in accordance with the assumptions required for valid causal inference.

### Statistical analysis

#### Descriptive and bivariate analysis

Descriptive statistics were used to summarise the characteristics of the sample and to estimate the prevalence (i.e., the proportion within the study sample) of perceived workplace discrimination and depressive symptoms overall and stratified by sex. These estimates are descriptive and not intended to represent population-level prevalence. Chi-square tests were used to explore bivariate associations between perceived workplace discrimination and depressive symptoms and to compare sociodemographic and occupational characteristics between men and women. These analyses were performed prior to conducting multivariable regression analyses.

#### Multivariate logistic regression

The associations between perceived workplace discrimination and depressive symptoms were assessed via logistic regression models. Initially, an unadjusted model was estimated for the full sample. Subsequently, sex-stratified models were fitted to explore potential effect modification by sex. To examine the relationship between discrimination and depressive symptom, the models were adjusted by controlling for sociodemographic and occupational variables, including age, company type, company tenure, educational level, job role, and shift work. A complete case analysis was conducted, in which individual participants (observations) were excluded from a given model if they had missing data on the exposure (perceived discrimination), outcome (depressive symptoms), or any of the covariates included in that model. This approach assumes that data are missing completely at random, meaning that the probability of missingness is unrelated to the values of the observed or unobserved data. No imputation was performed. The number of complete cases used for each analysis is reported in the corresponding tables.

#### Causal mediation analysis

A causal mediation analysis was conducted to evaluate the mediating role of psychological distress in the relationship between perceived workplace discrimination (exposure) and depressive symptoms (outcome). This analysis, was carried out using counterfactual-based methods, which allow decomposition of the total effect into direct and indirect components. Specifically, we estimated the natural indirect effect (NIE), which represents the portion of the total effect mediated through psychological distress, and the natural direct effect (NDE), which reflects the portion of the effect not operating through the mediator. We also estimated the total effect (TE) [30]. Finally, the proportion mediated (PM) was also calculated to estimate the relative contribution of the mediating pathway.

Causal interpretation of the NIE and NDE requires the following assumptions: (1) no unmeasured confounding between the exposure and the outcome; (2) no unmeasured confounding between the mediator and the outcome; (3) no unmeasured confounding between the exposure and the mediator; and (4) no mediator-outcome confounders that are affected by the exposure [30]. These assumptions were not formally evaluated using directed acyclic graphs, and their fulfilment cannot be guaranteed given the cross-sectional design of the study. However, covariates were selected based on theoretical plausibility and prior literature to reduce the risk of confounding. In addition, sensitivity analyses were conducted to assess the robustness of the mediation estimates under potential violations of these assumptions.

Given the binary nature of the outcome and the continuous nature of the mediator, and in order to meet the assumptions of the statistical procedure used, causal mediation analysis was performed using the *mediate* command in STATA version 18.0 [31], based on counterfactual approach. Two models were estimated: one for discrimination personally experienced by the worker and another for discrimination observed in the workplace. The first model included perceived discrimination (exposure), psychological distress (mediator), and depressive symptoms (outcome). The second model was additionally adjusted for age, sex, number of children, educational level, shift work, job role, and mining tenure. A probit model was specified for the binary outcome (moderate/severe depressive symptoms), and a linear model was used for the continuous mediator (psychological distress). Due to software constraints, it was not feasible to use a logit model for the binary outcome in combination with a linear mediator model; in this scenario, the *mediate* command in STATA requires a probit model. This approach is consistent with current recommendations when modelling a continuous mediator and a dichotomous outcome. The estimated effects (NIE, NDE and TE) are reported as odds ratios (OR) with robust 95% confidence intervals. The proportion mediated was also calculated. Statistical significance was determined using a threshold of *p* < 0.05.

#### Sensitivity analysis

A sensitivity analysis was conducted using E values to evaluate the robustness of the observed associations to potential unmeasured confounding factors. The E value represents the minimum strength of association that an unmeasured confounder would need to have with both the exposure and the outcome, beyond the measured covariates, to fully explain away the observed association [32]. This method is particularly useful in observational research, where residual confounding is always a concern. E values were computed via the *evalue* command in STATA [33]. While no strict threshold exists for E-value interpretation, higher values indicate more robust findings, whereas lower values suggest that even weak confounders might account for the observed effect. The full set of e-value results for logistic regression and mediation models is provided in Supplementary Material 2.

#### Post hoc power analysis

A post hoc power analysis was conducted for the female subgroup to assess the study’s ability to detect differences in depressive symptoms between women who reported experiencing workplace discrimination and those who did not. The analysis focused on both perceived and observed workplace discrimination and was based on the difference in the proportions of depressive symptoms between participants who reported and did not report experiencing (or observing) workplace discrimination. The power was calculated via STATA’s *power two proportions* command.

## Results

### Participants

The characteristics of the sample, which was composed of 712 mining sector workers, are presented in Table 1. The final analytical sample included 712 complete responses, exceeding the minimum required sample size. Most participants were employed by the principal company (70.93%), whereas 29.07% worked for subcontractors. The predominant marital status was married (52.53%), followed by single (34.55%). The majority of participants (83.15%) had children. Regarding to educational level, 37.08% held a university degree, followed by technical education (26.12%), secondary education (23.31%), postgraduate studies (12.22%), and primary education (1.26%). 66.01% of total workers were employed under a rotating shift system, with the 4 × 4 shift being the most common (57.99%), followed by the 7 × 7 (20.26%) and 4 × 3 (8.74%) shifts. In terms of job roles, 47.05% worked in the field, 35.11% performed mixed duties (field and administrative), and 17.84% held administrative positions exclusively. Regarding tenure with the company, 51.12% had worked for less than five years, whereas in the mining sector specifically, 45.37% reported 11–20 years of experience. Finally, 83.57% of workers reported being aware of antidiscrimination policies within the company, whereas 16.43% were unaware. The prevalence of perceived workplace discrimination was 13.56% (*n* = 95), that of observed workplace discrimination was 19.29% (*n* = 137), and depressive symptoms were present in 8.31% (*n* = 59) of the participants.

**Table 1:** Sociodemographic and occupational characteristics of the sample, stratified by sex (*N* = 712)

Variable	Men (*n* = 569)	Women (*n* = 143)	Total (*n* = 712)	*p* value
Age				< 0.001
Young workers (≤ 29 years)	10 (1.76)	7 (4.90)	17 (2.39)	
Mid-aged workers (30–44 years)	261 (45.87)	90 (62.94)	351 (49.30)	
Older workers (≥ 45 years)	298 (52.37)	46 (32.17)	344 (48.31)	
**Company type**				0.930
Principal company	404 (71.00)	101 (70.63)	505 (70.93)	
Contractor	165 (29.00)	42 (29.37)	207 (29.07)	
**Marital status**				< 0.001
Single	165 (29.00)	81 (56.64)	246 (34.55)	
Married	335 (58.88)	39 (27.27)	374 (52.53)	
Civil union	11 (1.93)	5 (3.50)	16 (2.25)	
Separated	23 (4.04)	6 (4.20)	29 (4.07)	
Divorced	32 (5.62)	12 (8.39)	44 (6.18)	
Widowed	3 (0.53)	0 (0)	3 (0.42)	
**Parental status**				< 0.001
No	68 (11.95)	52 (36.36)	120 (16.85)	
Yes	501 (88.05)	91 (63.64)	592 (83.15)	
**Educational level**				< 0.001
Primary/Secondary education	166 (29.17)	9 (6.29)	175 (24.58)	
Technical education	159 (27.94)	27 (18.88)	186 (26.12)	
University (degree/Postgraduate)	244 (42.88)	107 (74.83)	351 (49.30)	
**Shift work**				< 0.001
No	164 (28.82)	78 (54.55)	242 (33.99)	
Yes	405 (71.18)	65 (45.45)	470 (66.01)	
**Shift type**				< 0.001
4 × 3	25 (6.19)	16 (24.62)	41 (8.74)	
4 × 4	247 (61.14)	25 (38.46)	272 (57.99)	
5 × 2	28 (6.93)	3 (4.62)	31 (6.61)	
7 × 7	75 (18.56)	20 (30.77)	95 (20.26)	
6 × 3	19 (4.70)	0 (0)	19 (4.05)	
Other	10 (2.48)	1 (1.54)	11 (2.35)	
**Job role**				< 0.001
Administrative	79 (13.88)	48 (33.57)	127 (17.84)	
Fieldwork	298 (52.37)	37 (25.87)	335 (47.05)	
Mixed	192 (33.74)	58 (40.56)	250 (35.11)	
**Company tenure**				< 0.001
< 5 years	270 (47.45)	94 (65.73)	364 (51.12)	
5–10 years	111 (19.51)	26 (18.18)	137 (19.24)	
11–20 years	145 (25.48)	21 (14.69)	166 (23.31)	
> 20 years	43 (7.56)	2 (1.40)	45 (6.32)	
**Mining tenure**				< 0.001
< 5 years	62 (10.90)	52 (36.36)	114 (16.01)	
5–10 years	73 (12.83)	30 (20.98)	103 (14.47)	
11–20 years	270 (47.45)	53 (37.06)	323 (45.37)	
> 20 years	164 (28.82)	8 (5.59)	172 (24.16)	
**Awareness of anti-discrimination policy**				0.165
No	99 (17.40)	18 (12.59)	117 (16.43)	
Yes	470 (82.60)	125 (87.41)	595 (83.57)	
**Perceived workplace discrimination**				0.069
No	580 (86.44)	106 (18.28)	474 (81.72)	
Yes	91 (13.56)	24 (26.37)	67 (73.63)	
**Observed workplace discrimination**				0.243
No	544 (80.71)	101 (18.57)	443 (81.43)	
Yes	130 (19.29)	30 (23.08)	100 (76.92)	
**Depression**				0.004
No	618 (91.69)	112 (85.50)	506 (93.19)	
Yes	56 (8.31)	19 (14.50)	37 (6.81)	

Notes: Percentages may not total 100% due to rounding. “Shift work” refers to rotating shift systems. “Mixed” job roles include positions that combine administrative and fieldwork tasks. The total sample comprised 712 participants; however, owing to missing data, the analytical sample size varied slightly for specific variables: *n* = 671 for perceived workplace discrimination and *n* = 674 for observed workplace discrimination and depressive symptoms. P values were calculated via chi-square tests to assess differences between men and women across categories

### Associations between perceived workplace discrimination and depressive symptoms

As shown in Table 2, a significant difference in the prevalence of depressive symptoms was observed according to perceived discrimination status, as determined through a bivariate chi-square analysis. Among participants who did not report experiencing discrimination, 6.03% showed depressive symptoms, compared to 23.08% among those who did (*p* < 0.001).

Using logistic regression, in the crude association analysis for the total sample (Table 3), workers who perceived workplace discrimination had significantly greater odds of depressive symptoms (OR: 4.67; 95% CI: 2.57–8.47; *p* < 0.001). After adjusting for potential confounders, the association became stronger (OR: 5.17; 95% CI: 2.70–9.91; *p* < 0.001).

When stratified by sex, men who perceived discrimination had significantly higher odds of depressive symptoms in the crude model (OR: 5.92; 95% CI: 2.89–12.13; *p* < 0.001), with even stronger odds in the adjusted model (OR: 7.42; 95% CI: 3.27–16.79; *p* < 0.001) (Table 3). Among women, both the crude and adjusted models suggested a positive association. However, neither reached statistical significance.

### Associations between observed workplace discrimination and depressive symptoms

A similar bivariate association was found for witnessed workplace discrimination. Among workers who had not witnessed discrimination, 5.51% exhibited depressive symptoms, compared to 20.00% among those who had (Table 2).

According to logistic regression, in the crude model, observed workplace discrimination was significantly associated with increased odds of depressive symptoms (OR: 4.28; 95% CI: 2.43–7.54; *p* < 0.001). This association remained significant after adjustment.

**Table 2:** Prevalence of perceived and observed workplace discrimination by depressive symptom status

	No Depression	With Depression	Chi2	*p* value
	*N (%)*	*N (%)*		
**Perceived Workplace Discrimination**				
*No*	545 (93.97%)	35 (6.03%)	29.86	0.001
*Yes*	70 (76.92%)	21 (23.08%)		
**Observed Workplace Discrimination**				
*No*	514 (94.49%)	30 (5.51%)	28.89	0.001
*Yes*	104 (80.00%)	26 (20.00%)		

Notes: Distribution of depressive symptoms (PHQ-9 score ≥ 10) by the presence or absence of perceived and observed workplace discrimination. *P* values were calculated via chi-square tests. Sample sizes differ due to missing data: *n* = 671 for perceived workplace discrimination and *n* = 674 for observed workplace discrimination

**Table 3:** Crude and adjusted logistic regression models of workplace discrimination and depressive symptoms stratified by sex

	Model	Total OR (95% CI)	*p* value	Men OR (95% CI)	*p* value	Women OR (95% CI)	*p* value
**Perceived Workplace Discrimination**	crude	4.67 (2.57–8.47)	< 0.001	5.92 (2.89–12.13)	< 0.001	2.38 (0.80–7.10)	0.119
	adjusted	5.17 (2.70–9.91)	< 0.001	7.42 (3.27–16.79)	< 0.001	2.57 (0.77–8.56)	0.122
**Observed Workplace Discrimination**	crude	4.28 (2.43–7.54)	< 0.001	4.89 (2.46–9.73)	< 0.001	2.97 (1.07–8.27)	0.037
	adjusted	4.01 (2.20–7.31)	< 0.001	4.99 (2.38–10.47)	< 0.001	3.55 (1.06–11.81)	0.039

Notes: Crude and adjusted odds ratios (OR) with 95% confidence intervals (CI) from logistic regression models assessing the associations between workplace discrimination (perceived and observed) and depressive symptoms (PHQ-9 score ≥ 10). Models are presented for the total sample and stratified by sex. The adjusted models control for age, number of children, educational level, shift work, job role, and company tenure. Sample sizes differ due to missing data: *n* = 671 for perceived workplace discrimination and *n* = 674 for observed workplace discrimination

According to the sex-stratified models (Table 3), men who observed discrimination had greater odds of depressive symptoms in both the crude model (OR: 4.89; 95% CI: 2.46–9.73; *p* < 0.001) and the adjusted model (OR: 4.99; 95% CI: 2.38–10.47; *p* < 0.001). Among women, both the crude (OR: 2.97; 95% CI: 1.07–8.27; *p* = 0.037 and adjusted models (OR: 3.55; 95% CI: 1.06–11.81; *p* = 0.039) showed statistically significant associations.

### Mediation analysis

#### Perceived workplace discrimination

Table 4 shows the results of the causal mediation analysis evaluating psychological distress as a mediator. The natural indirect effect (NIE) was significant (OR: 3.53; 95% CI: 2.13–5.84; *p* < 0.001), indicating that perceived discrimination increases the likelihood of depressive symptoms through psychological distress. The natural direct effect (NDE) was not statistically significant (*p* = 0.317), indicating that the effect is primarily mediated. The total effect (TE) was significant (OR: 5.18; 95% CI: 2.68–10.01; *p* < 0.001). Psychological distress mediated 86.67% of the total association between perceived workplace discrimination and depressive symptoms (Table 4).

In the adjusted mediation model, after controlling for age, sex, number of children, educational level, shift work, job role, and tenure, the NIE (OR: 3.03; 95% CI: 1.98–4.64; *p* < 0.001) and the TE (OR: 4.91; 95% CI: 2.73–8.84; *p* < 0.001) were statistically significant, while the NDE was not statistically significant (*p* = 0.123). Psychological distress mediated 81.44% of the total association between perceived workplace discrimination and depressive symptoms (Table 4).

**Table 4:** Causal mediation analysis of psychological distress as a mediator between workplace discrimination and depressive symptoms

	ORc	95% CI	*p* value	PM (%)	ORa	95% CI	*p* value	PM (%)
	Perceived Workplace Discrimination							
*Natural Indirect Effect (NIE)*	3.53	2.13–5.84	< 0.001	86.67%	3.03	1.98–4.64	< 0.001	81.44%
*Natural Direct Effect (NDE)*	1.46	0.69–3.10	0.317		1.61	0.87–2.98	0.123	
*Total Effect (TE)*	5.18	2.68–10.01	< 0.001		4.91	2.73–8.84	< 0.001	
	**Observed Workplace Discrimination**							
*Natural Indirect Effect (NIE)*	2.29	1.59–3.31	< 0.001	67.60%	2.04	1.52–2.74	< 0.001	65.89%
*Natural Direct Effect (NDE)*	1.91	0.98–3.74	0.059		1.90	1.04–3.45	0.035	
*Total Effect (TE)*	4.37	2.31–8.27	< 0.001		3.89	2.20–6.87	< 0.001	

Notes: Causal mediation analysis presenting odds ratios (OR) with 95% confidence intervals (CI) for natural indirect effects (NIE), natural direct effects (NDE) and total effects (TE). The crude models are unadjusted; the adjusted models control for age, sex, number of children, educational level, shift work, job role, and company tenure. The analyses included participants with complete data on psychological distress (*n* = 671). PM (%) = proportion of the total effect mediated by psychological distress

#### **Observed workplace discrimination**

Similar mediation patterns were found for observed discrimination. The natural indirect effect (NIE) was statistically significant (OR: 2.29; 95% CI: 1.59–3.31; *p* < 0.001). The natural direct effect (NDE) was nearly significant (OR: 1.91; 95% CI: 0.98–3.74; *p* = 0.059). The total effect (TE) was statistically significant (OR: 4.37; 95% CI: 2.31–8.27; *p* < 0.001). and psychological distress mediated 67.60% of the total association between observed workplace discrimination and depressive symptoms (Table 4).

In the adjusted model, after controlling for age, sex, number of children, educational level, shift work, job role, and tenure, all three parameters remained statistically significant: the NIE, the NDE, and the TE. Psychological distress mediated 65.89% of the total association between observed workplace discrimination and depressive symptoms (Table 4).

## Discussion

In the context of increasing global awareness of the mental health consequences of workplace inequities, this study examined the associations between perceived and observed workplace discrimination and depressive symptoms among Chilean male and female mining workers. The findings indicate that both forms of discrimination are strongly associated with depressive symptoms, with psychological distress acting as a significant mediator. These results have important implications for occupational mental health interventions in extractive industries such as mining.

### Comparison with previous studies

Logistic regression analyses revealed a significant association between perceived workplace discrimination and moderate to severe depressive symptoms. Workers who reported having experienced discriminatory acts were approximately five times more likely to report moderate or severe depressive symptoms than those who did not. These findings are consistent with those of prior studies demonstrating the detrimental effects of workplace discrimination on mental health outcomes [10, 12–16, 34]. For example, a prospective cohort study revealed that individuals exposed to workplace discrimination were more than twice as likely to develop depressive disorders within six months, even after controlling for psychosocial and sociodemographic variables [34]. Although the present study is cross-sectional, the consistency of these findings with those of longitudinal research strengthens the plausibility of a causal relationship.

Similarly, an international study found that 62.5% of individuals diagnosed with major depressive disorder reported either experiencing or anticipating workplace discrimination due to their mental health status [35]. Importantly, experienced discrimination was independently associated with an increased risk of unemployment, even after adjusting for educational level and treatment history [35]. These findings are consistent with previous literature documenting the pervasive nature of workplace stigma and the pressing need for interventions that address both structural barriers and internalised stigma, although stigma itself was not directly assessed in our study.

Notably, in this study, the adjusted odds ratio increased from 4.67 to 5.46 after potential confounders such as age, sex, number of children, educational attainment, shift work, job role, and mining tenure were accounted for. This increase suggests a more robust association after adjustment and reinforces the independent relationship between workplace discrimination and depressive symptoms.

A further relevant finding is that observed workplace discrimination, witnessing discriminatory behaviour directed at others, was also significantly associated with increased odds of depressive symptoms. Employees who witnessed discriminatory acts were nearly four times more likely to report moderate or severe depressive symptoms. This finding is consistent with previous research indicating that bystanders to discrimination also experience adverse psychological effects. For example, a study reported that employees who witnessed discrimination had 50% higher odds of anxiety or depressive symptoms, lower job satisfaction, and higher absenteeism [11]. These results suggest that exposure to a discriminatory workplace climate may contribute to poor mental health not only for direct victims but also for those indirectly affected, possibly through shared psychosocial stressors and organisational risk factors.

### Sex-Specific implications

The findings revealed a stronger association between perceived workplace discrimination and depressive symptoms among men (adjusted OR = 7.47) compared to women (adjusted OR = 2.46).While the literature consistently supports a significant association between workplace discrimination and mental health outcomes, particularly depression, evidence on sex differences in this relationship remains limited and inconclusive. Most studies have focused predominantly on female populations or do not report sex-stratified results [10, 15, 34, 36–38], making direct comparisons difficult. As such, the present findings contribute novel insights by exploring potential sex-specific patterns in a male-dominated occupational setting.

One possible explanation for the attenuated association observed among women lies in the unique characteristics of the mining sector. Women who remain in this male-dominated environment may constitute a resilient cohort, having developed more robust coping strategies to navigate a hostile work culture. In support of this, women in the sample were generally younger, had fewer children, were more frequently single, had higher levels of education, and were less likely to work in field positions. These demographic and occupational factors may buffer the psychological impact of workplace discrimination.

Nonetheless, resilience in these environments does not necessarily confer psychological immunity. This resilience often comes at a psychological cost. A recent report described a range of survival strategies adopted by women in the mining sector, including behavioural masculinisation, emotional withdrawal, and overperformance in response to systemic violence [39]. Some women internalise beliefs equating femininity with weakness, leading them to suppress emotional expression or conceal aspects of their identity. Others adopt traditional gender roles strategically, known as the “object strategy”, to gain acceptance or ultimately leave the industry due to cumulative psychological strain. While these coping mechanisms may allow women to remain in the workforce, they often reflect a disproportionate emotional and occupational burden [39]. These findings underscore the urgent need for gender-transformative workplace policies and structural reforms that explicitly address the psychological burden associated with survival-based adaptation. However, caution is needed against equating gender equity efforts solely with numerical balance. A gender-integrated workforce does not necessarily guarantee a psychologically healthy environment. Prior research has shown that gender-integrated workplaces are associated with elevated psychological distress, even after accounting for psychosocial job conditions. This underscores the importance of addressing not only the proportion of men and women but also the quality of gender relations and the cultural norms that shape workplace dynamics [40].

This pattern aligns with previous research demonstrating that both interpersonal and organisational sexism in male-dominated industries significantly undermine women’s sense of belonging and contribute to elevated levels of stress, anxiety, and depression [41]. As suggested in previous literature, organisational-level dynamics may also influence women’s job satisfaction and exacerbate the psychological toll of survival-based strategies; however, this aspect was not directly assessed in our study.

It is important to acknowledge the potential impact of sample size differences, i.e., 569 men versus 143 women, on the statistical power to detect significant associations among women. However, beyond statistical considerations, gender-based differences in the perception, experience, and reporting of discrimination must also be considered. In male-dominated environments such as mining, women who remain employed may represent a “survivor cohort,” whereas those most affected by discrimination may have already exited the industry. Additionally, women may adopt alternative coping or reporting strategies shaped by socialisation, fear of retaliation, or normalisation of mistreatment. These dynamics may attenuate the observed associations and warrant further investigation through mixed-methods approaches.

This interpretation is supported by a recent systematic review that revealed that gender-based workplace harassment and violence are prospectively associated with depression and substance use across sexes. However, women reported significantly higher rates of victimisation, supporting the notion that those who remain in the mining workforce may be disproportionately exposed to psychological strain despite underreporting or adapting to hostile environments [42].

These findings also have implications for men, who may face unique barriers to recognising and addressing mental health concerns. Traditional masculine norms often discourage help-seeking and favour self-reliant or avoidant coping strategies [43, 44]. Consequently, men may resort to dysfunctional behaviours such as substance use or risk-taking. Notably, mental health programmes often lack gender-sensitive approaches, highlighting the need for interventions that account for both biological sex and gender norms, particularly in occupational settings [45].

Importantly, the gender mandates embedded within traditional mining culture do not exclusively affect women. Recent research in the Chilean mining sector has documented how prevailing masculinised norms can also constrain male workers. Qualitative evidence from interviews and focus groups conducted in this context revealed that many men felt excluded from inclusion efforts, expressed uncertainty regarding career prospects, and felt pressured to conform to a model of masculinity rooted in emotional suppression, total work availability, and stoicism. These perceptions were echoed by women, who observed discomfort, resistance, or confusion among male colleagues in response to organisational change. According to the authors of this study, traditional mining culture imposes restrictive expectations on men that not only undermine their mental well-being but also limit their engagement in transformation processes [46]. These insights emphasise the need to reframe inclusion initiatives as collective endeavours involving all genders, not as binary oppositions but as shared struggles within a gendered occupational system.

These adaptations may shape how women perceive and report discriminatory experiences, potentially contributing to the weaker statistical association between discrimination and depressive symptoms observed in this study. Rather than indicating lower vulnerability, this attenuation likely reflects the effect of survival-based coping strategies that modulate symptom expression or disclosure. In contrast, the stronger association observed among men may stem from less flexible coping resources and greater stigma surrounding mental health help-seeking, as noted in the gender norms literature. Collectively, these findings highlight the urgent need for gender-responsive mental health policies that not only consider differences in symptom expression and reporting but also address the structural and cultural constraints embedded within masculinised occupational settings such as mining.

### Mechanisms and psychological mediation

There is growing evidence that workers in the extractive and construction industries face elevated risks of psychosocial stressors, with work‒life conflict and role ambiguity being particularly salient [47]. Similar challenges have been documented among fly in fly out (FIFO) mining workers, who frequently report difficulties in balancing professional responsibilities with family life. The participants often described feeling as although they lived in “two worlds,” struggling to reintegrate upon returning home. Recurrent themes included missing important family events, relationship strain, and feelings of exclusion from household routines [48].

The findings of the present study underscore the central role of psychological distress as a mediator in the relationship between perceived workplace discrimination and depressive symptoms. In both models (perceived and observed discrimination), psychological distress accounted for over 65% of the total effect, reaching 81% for perceived discrimination. These results support the plausibility of a mediation pathway, although the cross-sectional design of this study warrants caution when drawing causal inferences.

This stress-mediated relationship has also been reported across various occupational sectors. For example, research in the Chinese banking sector has revealed that workplace toxicity, including bullying, ostracism, and incivility, significantly decreases productivity, with depression serving as a key mediating variable [49]. These findings resonate with those of the present study, underscoring the cross-sectoral relevance of psychological distress as a transmission mechanism.

Consistent with these findings, a study conducted among nurses in Iran demonstrated that workplace discrimination was positively associated with both job stress and depression, with job stress acting as a partial mediator [50]. These findings reinforce the idea that discriminatory work environments not only inflict direct psychological harm but also generate emotional strain that can precipitate mental health deterioration.

Similarly, research involving immigrants in Italy revealed that workplace discrimination was linked to poor mental health outcomes through both direct pathways and indirect mechanisms involving loneliness and life dissatisfaction [51]. These results further illustrate how structural and interpersonal exclusion within the workplace can undermine mental health through interconnected psychosocial channels.

Beyond occupational settings, evidence from the general population indicates that both daily and cumulative experiences of discrimination are associated with heightened threat appraisals and stronger negative emotional responses [52]. These patterns, characterised by evaluative threat and affective reactivity, act as mediators in the relationship between discrimination and a wide range of health outcomes, including mental illness and chronic disease. Although the observed indirect effects were modest, their consistency across studies suggests that daily psychological stress processes may accumulate over time, extending the impact of discrimination beyond the immediate context.

In line with these external findings, the mediation analysis in the present study revealed that the natural direct effect (NDE) of perceived discrimination on depressive symptoms was generally nonsignificant, indicating that the association operates primarily through psychological distress. In contrast, the adjusted model for observed discrimination yielded a significant NDE, suggesting that witnessing discrimination may independently affect mental health, possibly via mechanisms such as moral distress, empathic strain, or perceived injustice.

The results also highlight important distinctions between perceived and observed discrimination. While both were significantly associated with depressive symptoms, perceived discrimination showed a stronger indirect pathway through psychological distress, whereas observed discrimination exhibited a more pronounced direct association. These findings suggest that witnessing discrimination may activate different psychological mechanisms than directly experiencing it, an area that warrants further empirical investigation.

Taken together, these distinctions imply that workplace mental health interventions should be tailored to address both direct and vicarious exposure to discrimination. In addition to eliminating discriminatory practices, organisations should implement programmes that reduce emotional reactivity, strengthen individual coping mechanisms, and mitigate moral distress. Such strategies may be particularly beneficial in high-risk occupational environments such as extractive industries. Building on these findings, it becomes evident that psychological distress acts not merely as an intermediate variable but also as the principal psychological mechanism through which workplace discrimination leads to depressive symptoms. Therefore, reducing sources of psychological distress should be a core focus of workplace mental health initiatives, particularly in high-risk, high-stress environments such as the mining sector.

### Relevance for the mining sector and similar industrial settings

The findings of this study have clear practical implications for occupational health in the mining industry. Interventions should focus not only on reducing discriminatory behaviours but also on mitigating their psychological consequences. Given the large proportion of the association between discrimination and depressive symptoms that is mediated by psychological distress, strategies such as workplace helplines, peer support systems, and inclusive organisational policies could play a critical role in preventing adverse mental health outcomes.

Moreover, as observed discrimination exhibited a more pronounced direct association with depressive symptoms, targeted initiatives such as leadership training and team-based workshops to eliminate visible and normalised discriminatory behaviours may be especially effective. These approaches can help transform organisational culture while addressing both overt and subtle forms of exclusion.

There are several examples of workplace interventions that may help reduce mental health problems in mining contexts [53]. At the individual level, mindfulness programmes implemented in Poland were associated with reductions in anxiety and depressive symptoms, although adherence was low and the effects showed limited sustainability over time [54]. Similarly, in Australia, a digital application tailored to the mining industry, based on behavioural activation and mindfulness, was tested and demonstrated improvements in depressive and anxiety symptoms, along with reduced absenteeism and increased productivity among those who completed the programme, despite high dropout rates [55]. At the organisational level, the MATES in Mining programme stands out as an adaptation of a peer-based intervention originally developed in the Australian construction industry, focused on suicide prevention and mental health promotion. The programme comprises three progressive levels of training: (1) a general awareness session for all workers, (2) additional training for “connectors” who act as peers linking colleagues at risk to support services, and (3) an intensive Applied Suicide Intervention Skills Training (ASIST) for a smaller group of designated staff. This model is complemented by field officer support, case management, and a 24/7 telephone helpline. This programme has been implemented in Australian coal mines and complemented with supervisor training, demonstrating feasibility and high acceptability. Significant improvements were observed in workers’ and supervisors’ confidence to identify and support colleagues with mental health problems, as well as an increased perception of organisational commitment to psychological wellbeing [56]. These examples highlight the value of combining peer support with leadership training in highly male-dominated settings such as mining. Therefore, both individual- and organisational-level strategies appear to be viable in the mining sector, as long as they are carefully adapted to the cultural realities of male-dominated industries. Beyond interventions targeting mental health, recent evidence has synthesized what works in reducing workplace discrimination. A meta-analysis of 70 studies [57] found that passive interventions, such as short-term educational sessions or reminders of bias processes, were largely ineffective in changing behaviour. In contrast, interventions that directly target behaviour, by increasing individual accountability for decisions or by altering organisational social norms, were the most effective in reducing discriminatory practices. These findings highlight the importance of embedding anti-discrimination efforts within organisational structures and accountability systems, which is especially relevant in male-dominated industries such as mining, where both direct and observed discrimination may persist.

However, persistent barriers remain, including mental health stigma, limited awareness of available resources, and insufficient leadership engagement [53]. These challenges underscore the need for sustained, system-wide approaches that simultaneously address structural factors and individual vulnerabilities.

Building on these insights, it is crucial to recognise that effective interventions cannot rely solely on isolated or reactive initiatives. Instead, they should adopt a holistic, integrated approach that simultaneously addresses workplace hazards, organisational culture, and worker well-being. This perspective aligns with the Total Worker Health^®^ framework, which advocates for the integration of occupational safety and health protection with health promotion efforts to advance worker well-being comprehensively. In the mining sector, applying this integrated strategy could substantially strengthen mental health interventions by targeting both the elimination of discrimination and the reduction of psychosocial stressors at their root.

Importantly, given the high proportion of the association mediated by psychological distress, workplace policies must extend beyond the mere prevention of discriminatory acts. Robust psychosocial support systems are needed to buffer the emotional toll associated with working in high-risk, male-dominated environments such as mining. Programmes that proactively address occupational stress, burnout, and emotional isolation, particularly those that are culturally and gender sensitive, could significantly enhance worker well-being, resilience, and productivity. Embedding these interventions within a comprehensive organisational strategy would not only promote psychological safety but also contribute to the creation of more resilient, equitable, and sustainable work environments.

### Strengths and limitations

This study presents several notable strengths that enhance its methodological rigour and practical relevance. First, it addresses a highly relevant yet underexplored issue, the relationship between workplace discrimination and mental health, within the unique context of Chile’s large-scale mining sector. This setting provides original evidence from a critical but often understudied industrial environment. Second, the use of a counterfactual framework for causal mediation analysis represents a methodological advancement over traditional regression models, allowing for a more nuanced understanding of the mechanisms linking perceived workplace discrimination to depressive symptoms. The ability to decompose the total effect into direct and indirect pathways through psychological distress strengthens the interpretation of causal processes. Third, this approach offers actionable insights for occupational health by identifying a key modifiable mechanism that may be targeted through both preventive and therapeutic strategies. Fourth, the analysis accounted for a wide range of relevant sociodemographic and occupational variables, such as age, sex, education, job role, tenure, and shift work, enhancing the internal validity of the findings. Fifth, sex-stratified models provided more granular insight into potential gender differences in the impact of discrimination, informing the design of more tailored workplace health policies. Sixth, the application of advanced statistical techniques, including the use of STATA’s *mediate* command and e-value sensitivity analysis, demonstrates a rigorous implementation of modern epidemiological tools and reinforces the robustness of the results. In particular, the e-value analysis showed that the observed associations would require strong unmeasured confounding to be explained away, further strengthening the validity of the main findings (see Supplementary Material 2). Finally, the sample included 712 workers from both the principal company and subcontractors, exceeding the estimated minimum sample size and encompassing broad representativeness within the mining sector. Such diversity facilitates a more comprehensive understanding of discrimination experiences across job roles and employment types.

Despite these strengths, several limitations should be acknowledged. First, the cross-sectional design limits the ability to draw definitive causal conclusions among the relationship between perceived workplace discrimination, psychological distress, and depressive symptoms. Although advanced causal inference techniques were used to strengthen interpretation, the possibility of reverse causation and residual confounding cannot be eliminated.

Second, although the mediation analysis followed a counterfactual approach and incorporated theoretically relevant covariates, key assumptions for causal interpretation, such as the absence of unmeasured confounding between exposure, mediator, and outcome, were neither empirically tested nor assessed using directed acyclic graphs, due to limitations inherent in the study design. Third, the reliance on self-reported data introduces the potential for information bias, including social desirability or recall bias, particularly in workplace environments where concerns about retaliation may influence disclosure. Fourth, generalizability may be limited given the focus on a single large-scale mining company in Chile; findings may not be applicable to other industries or cultural contexts. Finally, the underrepresentation of women in the sample reduced the statistical power to detect meaningful subgroup differences. Post hoc power analyses revealed limited power to detect differences in depressive symptoms among women exposed to perceived (23.15%) and observed (42.36%) discrimination, increasing the risk of type II error and suggesting that nonsignificant findings in this subgroup should be interpreted with caution.

Future studies should seek to overcome these limitations by employing longitudinal designs, ensuring greater gender balance in sampling, and empirically verifying causal assumptions via formal directed acyclic graphs based approaches. Additionally, qualitative components or mixed-methods approaches should be considered to capture the complexity and nuance of discriminatory experiences more comprehensively.

## Conclusion

This study provides robust empirical evidence on the association between perceived workplace discrimination and depressive symptoms among workers in Chile’s large-scale mining sector. The findings indicate that both perceived and observed workplace discrimination are significantly associated with an increased likelihood of depressive symptoms, with this association being stronger among men than women. These sex-specific differences may reflect contextual and cultural factors within the mining industry, including traditional gender norms, reporting barriers, and survival strategies more commonly adopted by women.

Moreover, causal mediation analyses revealed that a substantial proportion of this association is explained by psychological distress, suggesting that the impact of workplace discrimination on mental health primarily operates through emotional and psychological mechanisms. This finding highlights the importance of considering psychological distress not only as an outcome but also as a key mediating pathway linking discriminatory work environments to mental health disorders such as depression.

Collectively, these results highlight the urgent need for organisational policies to reduce workplace discrimination and foster psychologically safe environments within the mining industry. In parallel, occupational health strategies should incorporate targeted emotional support and psychological interventions, particularly in highly masculinised and high-risk labour settings such as mining.

## Supplementary Information

Supplementary Material 1

Supplementary Material 2

## Data Availability

The datasets generated and/or analysed during the current study are not publicly available due to confidentiality agreements with the participating mining company but are available from the corresponding author upon reasonable request.

## Abbreviations

PHQ-9: Patient Health Questionnaire-9
K6: Kessler Psychological Distress Scale
NIE: Natural Indirect Effect
NDE: Natural Direct Effect
TE: Total Effect
PM: Proportion Mediated
