# Supplementary material for: Association between perceived discrimination and depressive symptoms among male and female mining workers in Chile: a sex-stratified analysis and the mediating role of psychological distress
Source: BMC Public Health. 2026 Jan 21;26:274. doi: 10.1186/s12889-025-25787-2 (PMC12825288; doi:10.1186/s12889-025-25787-2)
Supplement: Supplementary file 1 — Supplementary Material 1 [file 12889_2025_25787_MOESM1_ESM.docx]

**Supplementary Material 1: Perceived Workplace Discrimination Questionnaire**

**Prevalence of Observed Workplace Discrimination (discrimination directed at others that you have observed)**

*For the purposes of this study, workplace discrimination is defined as any unfair or negative treatment toward employees based on individual characteristics or social group membership, which is not related to job performance.*

1. In the past year, have you observed acts of discrimination against other people in your workplace?

- Yes, I have observed such acts
- No, I have not observed such acts

**Reasons for Observed Workplace Discrimination**

*For the purposes of this study, workplace discrimination is defined as any unfair or negative treatment toward employees based on individual characteristics or social group membership, which is not related to job performance.*

2. Please indicate the reason(s) for the discrimination you have witnessed or observed against others in your workplace (you may select more than one option):

- Sex/Gender
- Sexual orientation
- Age
- Migrant status
- Ethnicity/Race
- Physical appearance
- Disability
- Socioeconomic level
- Educational level or institution
- Other (please specify)

**Prevalence of Perceived Workplace Discrimination (discrimination directed at you personally)**

*For the purposes of this study, workplace discrimination is defined as any unfair or negative treatment toward employees based on individual characteristics or social group membership, which is not related to job performance.*

3. In the past year, have you felt discriminated against in your workplace?

- Yes, I have felt discriminated against/a
- No, I have not felt discriminated against

**Reasons for Perceived Workplace Discrimination**

*For the purposes of this study, workplace discrimination is defined as any unfair or negative treatment toward employees based on individual characteristics or social group membership, which is not related to job performance.*

4. Please indicate the reason(s) you believe led to your experience of discrimination in your workplace (you may select more than one option):

- Sex/Gender
- Sexual orientation
- Age
- Migrant status
- Ethnicity/Race
- Physical appearance
- Disability
- Socioeconomic level
- Educational level or institution
- Other (please specify)

**Perpetrator of Discrimination**

*Please indicate your level of agreement with the following statements:*

5. Acts of discrimination in your workplace (either directed at you or observed) were committed by co-workers

- Strongly disagree
- Disagree
- Agree
- Strongly agree

6. Acts of discrimination in your workplace (either directed at you or observed) were committed by direct or indirect supervisors.

- Strongly disagree
- Disagree
- Agree
- Strongly agree

7. Acts of discrimination in your workplace (either directed at you or observed) were committed by external persons (e.g., visitors, external consultants, suppliers, inspectors).

- Strongly disagree
- Disagree
- Agree
- Strongly agree

**Situations in Which Discrimination Occurred**

*Please indicate your level of agreement with the following statements:*

8. Acts of discrimination in your workplace (either directed at you or observed) occurred during formal situations (e.g., scheduled meetings, performance evaluations, training sessions)

- Strongly disagree
- Disagree
- Agree
- Strongly agree

9. Acts of discrimination in your workplace (either directed at you or observed) occurred during informal situations (e.g., break room conversations, company sports activities, company celebrations)

- Strongly disagree
- Disagree
- Agree
- Strongly agree

**Frequency of Discrimination**

*Please indicate how often the acts occurred:*

10. How frequently have acts of discrimination occurred in your workplace (either directed at you or observed)?

- A few times a year
- A few times a month
- A few times a week
- Daily

**Type of Discrimination**

*Please indicate your level of agreement with the following statements:*

11. Acts of discrimination in your workplace (either directed at you or observed) mainly involved physical aggression (e.g., being hit, pushed, kicked).

- Strongly disagree
- Disagree
- Agree
- Strongly agree

12. Acts of discrimination in your workplace (either directed at you or observed) mainly involved psychological aggression (e.g., being yelled at, insulted, threatened, deliberately ignored, discredited in front of others, unfairly accused, mocked, or intentionally overloaded with unmanageable tasks)

- Strongly disagree
- Disagree
- Agree
- Strongly agree

**Effects of Discrimination**

*Please indicate how these acts affected your health and job performance:*

13. Acts of discrimination in your workplace (either directed at you or observed) affected your health in the following way:

- No effect
- Mild
- Severe
- Very severe

13b. If your health has been affected, please specify what health problems you have experienced.

________________________________________________________________________________________________________________________________________________________________________________________________________________________________________________________________________

14. Acts of discrimination in your workplace (either directed at you or observed) affected your job performance in the following way:

- No effect
- Mild
- Severe
- Very severe

14b. If your job performance has been affected, please specify what performance issues you have experienced.

________________________________________________________________________________________________________________________________________________________________________________________________________________________________________________________________________
