# Supplementary material for: Association between perceived discrimination and depressive symptoms among male and female mining workers in Chile: a sex-stratified analysis and the mediating role of psychological distress
Source: BMC Public Health. 2026 Jan 21;26:274. doi: 10.1186/s12889-025-25787-2 (PMC12825288; doi:10.1186/s12889-025-25787-2)
Supplement: Supplementary file 2 — Supplementary Material 2 [file 12889_2025_25787_MOESM2_ESM.docx]

**Supplementary Material 2. E-values and Interpretation for Logistic Regression and Mediation Analyses of the Association Between Workplace Discrimination and Depressive Symptoms**

**Logistic Regression Analysis**

The logistic regression results revealed strong and statistically significant associations between workplace discrimination, both perceived and observed, and the presence of moderate to severe depressive symptoms among mining workers. In the total sample, workers who reported perceived workplace discrimination (PWD) had 5.17 times higher odds of experiencing depressive symptoms (95% CI: 2.70–9.91). This association was accompanied by an E-value of 9.831, indicating that an unmeasured confounder would need to be associated with both the exposure and the outcome by a risk ratio of at least 9.8 to fully explain away the observed effect, thereby demonstrating the robustness of this finding.

Among men, the association was even stronger (OR = 7.42; 95% CI: 3.27–16.79), with an E-value of 14.323 and an E-value of 6.013 for the lower limit of the confidence interval, indicating that only a very strong unmeasured confounder could nullify the observed association. In contrast, among women, although the association remained positive (OR = 2.57), it was not statistically significant (95% CI: 0.77–8.56), and the E-value for the lower confidence limit was 1.000, suggesting that even a relatively weak confounder could account for the association, potentially due to the smaller sample size in this subgroup.

Regarding observed workplace discrimination (OWD), statistically significant associations were also observed. In the total sample, workers who witnessed discrimination had an adjusted OR of 4.01 (95% CI: 2.20–7.31), with an E-value of 7.497 and a lower-bound E-value of 3.832, reinforcing the robustness of this association. Among men, the OR was 4.99 (95% CI: 2.38–10.47; E-value = 9.467; lower bound = 4.200), and among women, 3.55 (95% CI: 1.06–11.81; E-value = 6.566; lower bound = 1.340), suggesting that witnessing discrimination is also a significant predictor of depressive symptoms, especially among women where the association reached statistical significance despite the smaller subgroup.

**Mediation Analysis**

The mediation models provide clear evidence that psychological distress is a key mechanism linking workplace discrimination to depressive symptoms. For perceived discrimination, the natural indirect effect (NIE) was statistically significant (OR = 3.03; 95% CI: 1.98–4.64), with an E-value of 5.526 and a lower-bound E-value of 3.389, indicating a strong and robust mediating effect. The natural direct effect (NDE), however, was not statistically significant (OR = 1.61; 95% CI: 0.87–2.98; E-value = 2.619; lower bound = 1.000), suggesting that most of the total effect (TE = 4.91; 95% CI: 2.73–8.84; E-value = 9.305; lower bound = 4.912) operates through increased psychological distress.

For observed discrimination, both the indirect (NIE = 2.04; 95% CI: 1.52–2.74; E-value = 3.515; lower bound = 2.430) and direct effects (NDE = 1.90; 95% CI: 1.04–3.45; E-value = 3.211; lower bound = 1.270) were statistically significant. This suggests that witnessing discrimination affects mental health not only through emotional distress but also via direct pathways, potentially related to perceptions of injustice, moral injury, or deterioration in the workplace climate. The total effect (TE = 3.89; 95% CI: 2.20–6.87; E-value = 7.257; lower bound = 3.841) further supports the relevance of both mediated and unmediated mechanisms.

Together, these findings, supported by consistently high E-values, confirm that the associations between workplace discrimination and depressive symptoms are unlikely to be explained by unmeasured confounding. They underscore the need for comprehensive organizational interventions that target not only discriminatory practices but also the emotional and psychological consequences of working in high-stress, male-dominated environments such as the mining sector.

**Table 1. E-values for logistic regression models and causal mediation analysis of the association between workplace discrimination and depressive symptoms**

| **Analysis Type** | **Evaluated Association** | **Adjusted OR (95% CI)** | **E-value (OR)** | **E-value (Lower CI)** |
| --- | --- | --- | --- | --- |
| *Logistic Regression* | PWD → Depressive symptoms (total sample) | 5.17 (2.70 – 9.91) | 9.831 | 4.851 |
| *Logistic Regression* | PWD → Depressive symptoms (men) | 7.42 (3.27 – 16.79) | 14.323 | 6.013 |
| *Logistic Regression* | PWD → Depressive symptoms (women) | 2.57 (0.77 – 8.56) | 4.598 | 1.000 |
| *Logistic Regression* | OWD → Depressive symptoms (total sample) | 4.01 (2.20 – 7.31) | 7.497 | 3.832 |
| *Logistic Regression* | OWD → Depressive symptoms (men) | 4.99 (2.38 – 10.47) | 9.467 | 4.200 |
| *Logistic Regression* | OWD → Depressive symptoms (women) | 3.55 (1.06 – 11.81) | 6.566 | 1.340 |
| *Mediation Analysis* | PWD → Depressive symptoms (Natural Indirect Effect - NIE) | 3.03 (1.98 – 4.64) | 5.526 | 3.389 |
| *Mediation Analysis* | PWD → Depressive symptoms (Natural Direct Effect - NDE) | 1.61 (0.87 – 2.98) | 2.619 | 1.000 |
| *Mediation Analysis* | PWD → Depressive symptoms (Total Effect - TE) | 4.91 (2.73 – 8.84) | 9.305 | 4.912 |
| *Mediation Analysis* | OWD → Depressive symptoms (Natural Indirect Effect - NIE) | 2.04 (1.52 – 2.74) | 3.515 | 2.430 |
| *Mediation Analysis* | OWD → Depressive symptoms (Natural Direct Effect - NDE) | 1.90 (1.04 – 3.45) | 3.211 | 1.270 |
| *Mediation Analysis* | OWD → Depressive symptoms (Total Effect - TE) | 3.89 (2.20 – 6.87) | 7.257 | 3.841 |

**Note**: PWD = Perceived Workplace Discrimination; OWD = Observed Workplace Discrimination; OR = Odds Ratio; CI = Confidence Interval; NIE = Natural Indirect Effect; NDE = Natural Direct Effect; TE = Total Effect. E-values represent the minimum strength of association that an unmeasured confounder would need to have with both the exposure and the outcome, conditional on the measured covariates, to fully explain away the observed association. Higher E-values indicate greater robustness to potential unmeasured confounding. E-values are reported for the adjusted odds ratios and for the lower bound of their 95% confidence intervals.
